# Direct Observation of Tantalum Nitride Photoelectrode Degradation by Environmental Transmission Electron Microscopy

**DOI:** 10.1021/acsnanoscienceau.6c00123

**Published:** 2026-09-26

**Authors:** Annett Th⌀gersen, Shima Kadkhodazadeh, Michael Stanislaus Seifner, Monia Runge Nielsen, Ingvild J. T. Jensen, Martin Fleissner Sunding, Ingeborg-Helene Svenum, Mathieu Grandcolas, Øystein Dahl, Murat Nulati Yesibolati, Thomas Willum Hansen, Athanasios Chatzitakis

**Affiliations:** † 275243 SINTEF Industry, Materials Physics, Forskningsveien 1, Oslo 0373, Norway; ‡ DTU Nanolab., 5205 Technical University of Denmark, Fysikvej, Kgs. Lyngby 2800, Denmark; § Centre for Materials Science and Nanotechnology, Department of Chemistry, 6305 University of Oslo, Gaustadalléen 21, Oslo NO-0349, Norway

**Keywords:** TEM, PEC, ETEM, photocorrosion, Ta_3_N_5_, environmental TEM, water splitting

## Abstract

Stability under realistic operating conditions remains a critical challenge for photoelectrochemical (PEC) materials in solar fuel production, where photocorrosion often leads to rapid performance degradation. In particular, the formation of insulating amorphous TaO_*x*_/Ta­(O,N)_*x*_ surface interphases is widely recognized as a key factor limiting charge transfer and photocurrent stability in Ta_3_N_5_ photoanodes. Addressing this challenge requires experimental approaches capable of probing dynamic processes at the atomic level under relevant operating conditions. Here, we develop an *in situ* environmental transmission electron microscopy (ETEM) platform that integrates simultaneous light illumination, electrical biasing, and controlled water vapor exposure within a commercially available chip, supplemented with a customized optical add-on. This multistimulus configuration enables real-time atomic-scale imaging of PEC materials under conditions closely resembling operation. Applying this platform to Ta_3_N_5_, we directly capture its structural evolution under combined stimuli and reveal a previously unresolved degradation pathway involving nanocrystal detachment, migration, and inward-propagating amorphization. These processes lead to the growth of amorphous surface layers that are directly associated with performance loss in operating photoanodes. More broadly, this work establishes a mechanistic framework linking nanoscale structural transformations to macroscopic device degradation, providing concrete targets for stabilization strategies in Ta_3_N_5_ and related metastable PEC materials. In addition, the results demonstrate the broader potential of multistimulus ETEM for investigating complex degradation phenomena in functional energy materials.

## Graphical abstract

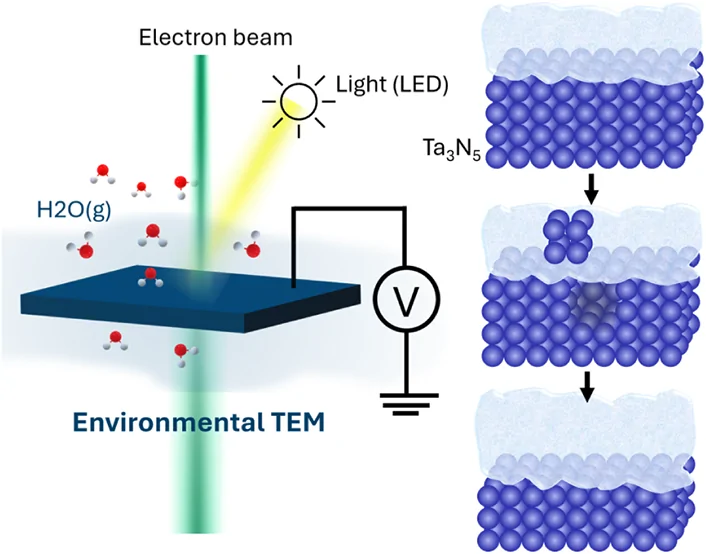

## Introduction

Achieving a sustainable energy future requires technologies that can efficiently and durably convert solar energy into chemical fuels. Photoelectrochemical (PEC) water splitting offers a direct route to produce hydrogen, a clean and versatile energy carrier.^1^ However, progress in PEC water splitting hinges not only on enhanced performance, but also on improving long-term stability of the materials involved. Among candidate photoanode materials, tantalum nitride (Ta_3_N_5_) stands out due to its suitable bandgap (∼2.1 eV) for visible light absorption and favorable band alignment with water redox potentials.^2–6^ Theoretically and assuming an external quantum efficiency (EQE) of 100%, Ta_3_N_5_ can deliver a solar-to-hydrogen (STH) conversion efficiency of 15.25%.^7–10^

Despite these advantages, Ta_3_N_5_ suffers from severe stability limitations under operational conditions, commonly attributed to photocorrosion through surface oxidation.^11,12^ This degradation is typically accompanied by the formation of amorphous TaO_*x*_/Ta­(O,N)_*x*_ surface interphases, which act as electronically resistive layers that hinder interfacial charge transfer and lead to rapid photocurrent decay during operation. Consequently, improving the stability of Ta_3_N_5_ is not only a materials challenge but a key bottleneck for achieving sustained device performance. Typical operating conditions for Ta_3_N_5_ photoanodes involve anodic potentials on the order of ∼1.0 V vs Reversible Hydrogen Electrode (RHE), under which photocorrosion is known to accelerate.

Understanding how operational conditions drive degradation at the atomic and nanoscale is therefore essential for developing effective stabilization strategies. Previous studies, including our *operando* ambient-pressure X-ray photoelectron spectroscopy (AP-XPS) work, have revealed electronic instabilities such as Fermi-level pinning and highlighted the importance of surface chemistry in controlling oxidation behavior.^7,13^ However, the nanoscale structural pathways by which crystalline Ta_3_N_5_ transforms into these amorphous, performance-limiting interphases under realistic operating conditions remain largely unexplored. This lack of mechanistic understanding limits the ability to rationally design stabilization strategies, forcing reliance on empirical approaches to surface modification and protection.

Addressing this challenge requires experimental platforms that can simultaneously apply multiple PEC-relevant stimuli while maintaining atomic-resolution imaging. Here, we introduce an *in situ* environmental transmission electron microscopy (ETEM) approach that integrates controlled water vapor exposure, electrical biasing, and localized light illumination within a single experimental configuration. ETEM uniquely allows specimens to be exposed to controlled gas environments, such as water vapor, while maintaining atomic-resolution imaging.^14,15^ To replicate biasing and light illumination, Ta_3_N_5_ thin films were mounted on Protochips focused ion beam (FIB)-optimized E-chips^16^ and illuminated using a millimeter-scale light-emitting diode (LED) positioned in front of the sample. This multistimulus configuration provides access to real-time observation of dynamic structural processes and their correlation to the applied environmental triggers.

Using Ta_3_N_5_ as a model system, we apply this platform to track its structural evolution under combined stimuli. The experiments demonstrate its capability to directly correlate multiple environmental inputs with nanoscale transformations, addressing a key limitation in the field where previous studies have typically examined water-mediated reactions with only one or two stimuli.^15,17–19^ More broadly, by linking nanoscale structural dynamics to macroscopic degradation pathways, the developed ETEM framework establishes a versatile methodology for investigating functional materials under realistic environments and provides actionable insight into the design of more stable PEC systems and related energy materials.

## Results

A Ta_3_N_5_ film deposited on a fused silica substrate is investigated in this work. Scanning electron microscopy (SEM) images of the sample reveal a polycrystalline morphology composed of nanoscale grains (Figure 1A). Cross-sectional scanning transmission electron microscopy (STEM) analysis shows that the film is approximately 600 nm thick and contains some level of porosity (Figure 1B). High-resolution STEM images and their corresponding fast Fourier transforms (FFT, location shown with red dashed square) confirm the polycrystalline nature of the Ta_3_N_5_ film, as well as the presence of a ∼1.5 nm thick amorphous surface layer (Figure 1C,D). Energy-dispersive X-ray spectroscopy (EDS) mapping of the surface (Figure 1E) and the corresponding line scan (Figure 1F) reveal oxygen enrichment and nitrogen depletion within this amorphous surface region relative to the bulk Ta_3_N_5_. These observations are consistent with the spontaneous formation of a thin native surface oxide or oxynitride layer, likely comprising TaO_*x*_ and/or Ta­(O,N)_*x*_ species, following exposure to ambient conditions during sample handling and transfer. The increase in N signal in the line scan reflects the transition from vacuum into the Ta_3_N_5_ film, where nitrogen is present. Variations in the relative Ta intensity near the surface are attributed to the presence of the oxide layer, where N depletion and O enrichment alter the relative EDS signal intensities, giving rise to an apparent increase in the Ta signal rather than a true enrichment of Ta.

**Figure 1:**
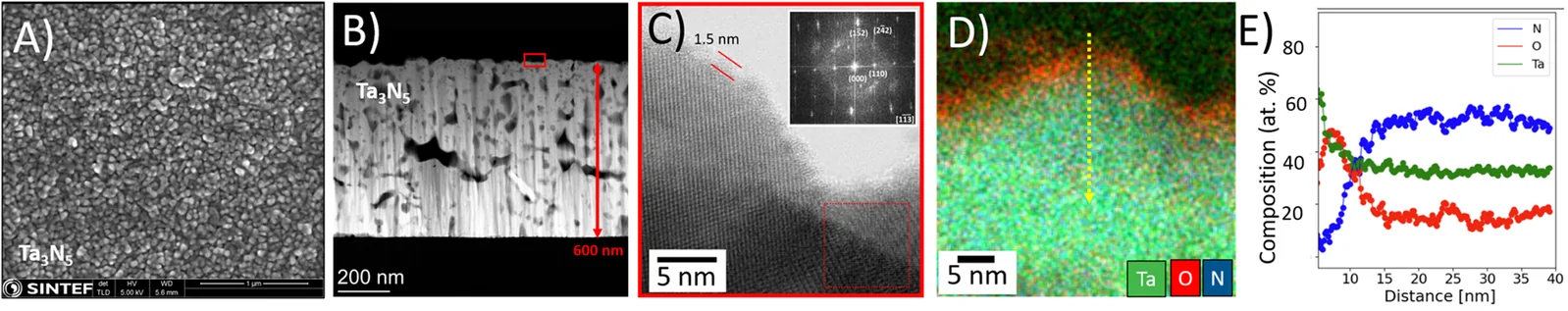
A) An overview SEM image of the Ta_3_N_5_ sample. B) Low-magnification dark filed STEM image of the sample’s cross-section. C) High-resolution bright filed STEM image of the Ta_3_N_5_ surface, with FFT pattern as inset (location as red dashed square), revealing the <113> zone-axis of the grain. D) EDS elemental map of the Ta_3_N_5_ surface. E) The corresponding line scan profile along the yellow line in (D).

### *In Situ* TEM Setup

A schematic of the multistimulus *in situ* experimental setup is shown in Figure 2A, while Figure 2B illustrates the customized Protochips Fusion Select TEM biasing holder with the E-chip and integrated light source. Thin lamellae of Ta_3_N_5_ were prepared using a plasma focused ion beam (FIB) and mounted on E-chips following a standard TEM preparation protocol, including deposition of a protective Pt layer (Figure 2C). Local Pt deposition was then used to establish electrical contact between the sample and the E-chip electrodes. Two geometries were fabricated: (i) a continuous Pt layer on Ta_3_N_5_ and (ii) a U-shaped cut through the Pt and Ta_3_N_5_ layers (Figure 2D,E). The latter promotes current flow across different layers and better replicates the intended PEC anode configuration.^20^ Details of the FIB preparation are provided in the Supporting Information (SI), Section I.

**Figure 2:**
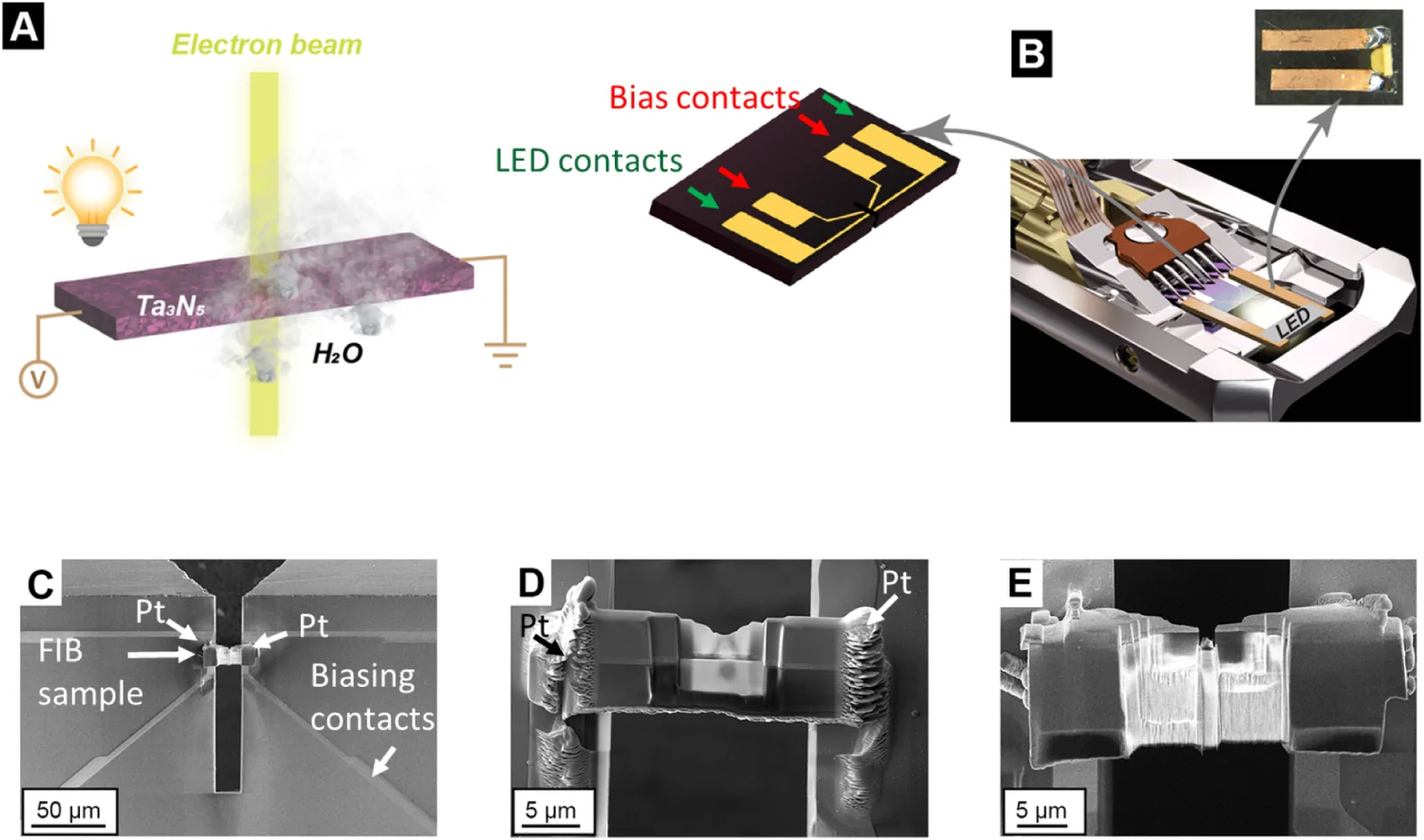
A) Schematic of the *in situ* setup combining light illumination, electrical biasing, and water vapor exposure. B) Customized Fusion Select Protochips biasing TEM holder (adapted with permission from ref 21 Copyright 2026 [Protochips]) with E-chip (bias from the inner contacts) and LED (connected to the outer contacts). C) SEM image of a thin-film TEM sample mounted on the biasing chip. D) FIB-prepared Ta_3_N_5_ sample with continuous Pt layer. E) FIB-prepared Ta_3_N_5_ sample with U-shaped geometry.

To enable *in situ* illumination, millimeter-scale light-emitting diodes (LEDs)^22^ soldered onto thin Cu foil strips were integrated into the biasing holder, inspired by strategies reported previously.^23^ In our configuration, the LED was mounted separately from the E-chip, with electrical contacts aligned above the chip’s electrode pads, allowing light control via the holder without additional FIB steps. The white LED has (ample) light output at wavelength of 460 nm, allowing for e-h pair generation/band-to-band absorption in Ta_3_N_5_. This design simplifies assembly and enables simultaneous application of water vapor, electrical bias, and light during ETEM imaging (Figure 2B; details in SI, Figure S2 Section II).

The experimental sequence began with imaging under vacuum to identify suitable regions. The sample was then exposed to 3 mbar H_2_O­(g) for 5 min while continuously recording one region and shielding others to minimize beam effects. After evacuation, images were acquired. Subsequent steps included: (i) water vapor + light illumination (LED bias 3.5 V, 5 min), (ii) water vapor +1.1 V electrical bias (5 min), and (iii) combined water vapor, light, and bias for 10 min, followed by imaging under vacuum. Finally, dynamic imaging was performed during simultaneous application of all stimuli. A parallel experiment followed the same protocol but with continuous beam exposure. Additional experiments and current/bias profiles are provided in the SI-III.

### Surface Reaction of Ta_3_N_5_

Successive TEM images of the same region of the FIB lamella with a continuous Pt layer on Ta_3_N_5_, under varying conditions are shown in Figure 3A–D: (A) high vacuum, (B) after water vapor exposure (3 mbar), (C) after water vapor and light illumination, and (D) after water vapor, light, and 1.1 V bias. Both beam-on (continuous electron beam exposure) and beam-off (only exposed to the electron beam without other stimuli) conditions are presented. These images reveal progressive morphological changes in response to the applied stimuli. Notably, Figure 3D shows significant structural modifications, including a pronounced thickening of the amorphous surface layer. Quantitative analysis of the amorphous layer area (Figure 4) confirms this trend under both beam-on and beam-off conditions (details in SI). Under beam-off conditions, the amorphous layer grows most rapidly when all three stimuli are applied simultaneously, consistent with previous reports that Ta_3_N_5_ degradation is a *synergistic process*, where combined photoexcitation and electrochemical potential in a humid environment accelerate amorphization.^7,24^

**Figure 3:**
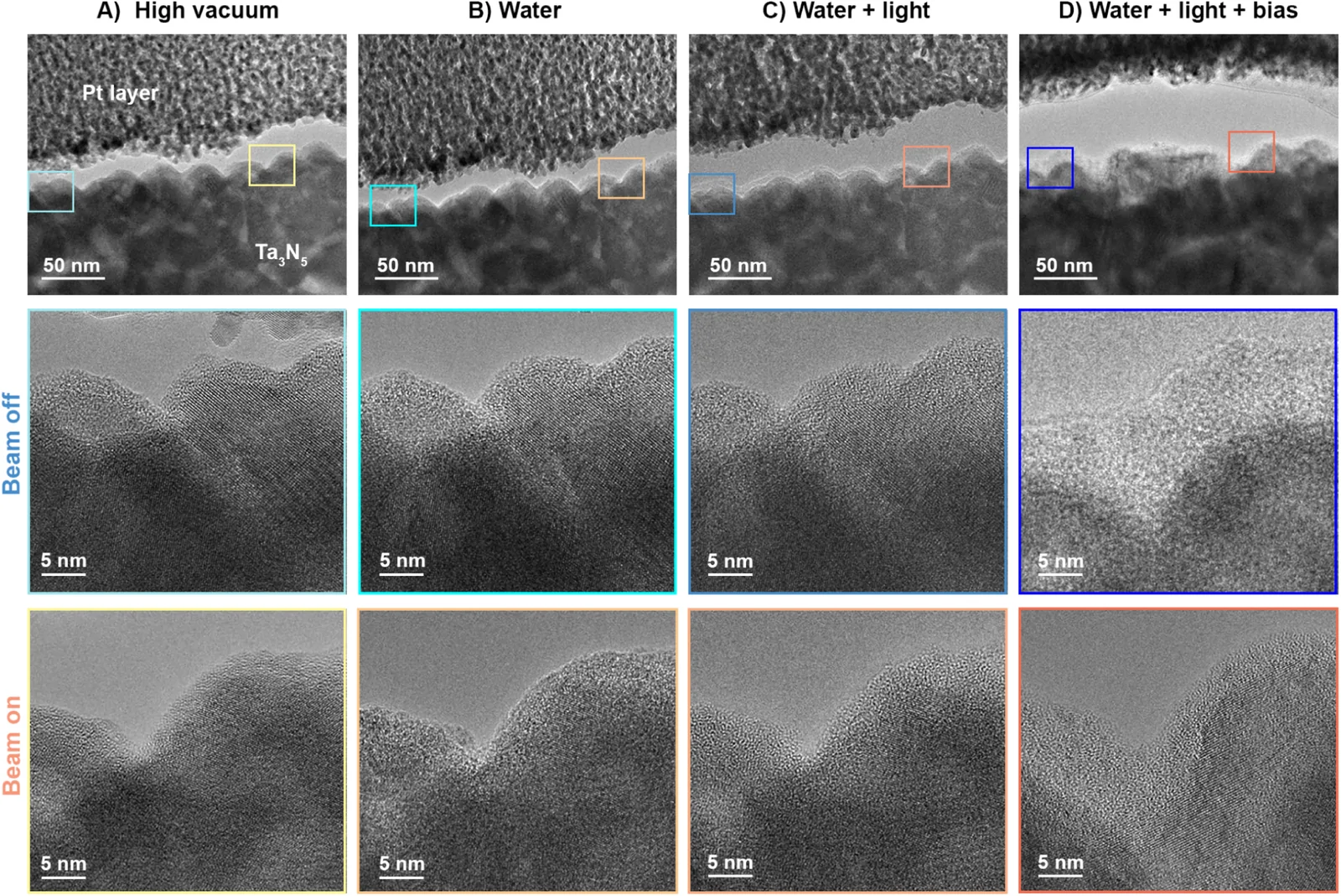
Medium- and high-magnification TEM images of the Ta_3_N_5_ sample under different conditions: A) high vacuum at the start of the experiment, B) after exposure to 3 mbar water vapor, C) after water vapor and light illumination, and D) after water vapor, light, and 1.1 V bias. Regions continuously imaged during exposure are labeled “beam on,” while those imaged only after exposure are labeled “beam off.”

**Figure 4:**
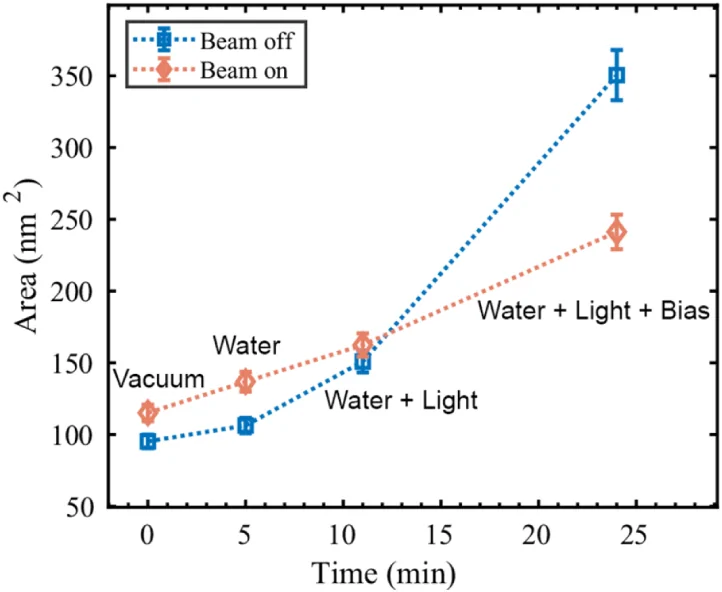
Calculated amorphous surface layer area from high-magnification TEM images in Figure 3 as a function of time and applied conditions.

Continuous *in situ* imaging (see SI video) further reveals a highly dynamic interface between crystalline Ta_3_N_5_ and the amorphous layer. Small nanocrystals (1–2 nm), with lattice spacings consistent with Ta_3_N_5_, detach from the interface between the native amorphous surface layer and the underlying crystalline bulk, and migrate within the amorphous matrix (Figure 5). Figure 5A shows a surface region under continuous beam exposure with corresponding FFT pattern, while Figure 5B highlights a detached nanocrystal confirmed as Ta_3_N_5_ by FFT analysis. Additional details of the expulsion process are provided in the SI and video, where the amorphous layer appears to stretch and expand as nanocrystals move; in some cases, these nanocrystals appear to become amorphous after reaching the surface. Although these observations were consistent across multiple regions, electron beam effects cannot be excluded. The role of biasing in this geometry is expected to be limited due to the continuous Pt layer covering Ta_3_N_5_. In contrast, the U-shaped geometry (Figure 6), exhibited much more pronounced degradation, where surface corrosion occurred even under water vapor and electron beam exposure alone.

**Figure 5:**
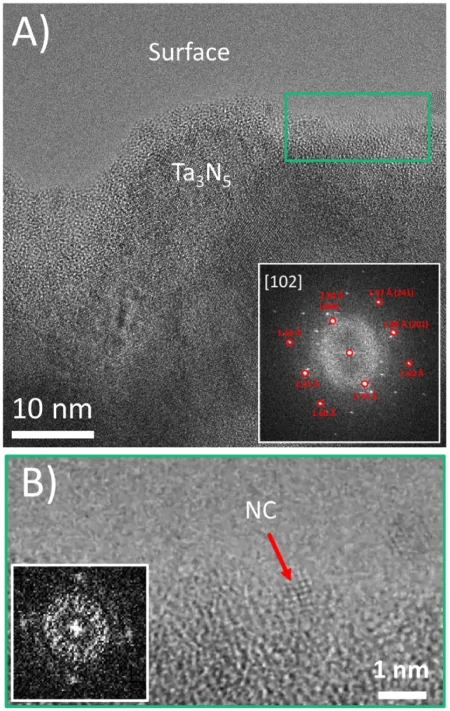
A) TEM image of the investigated area with the FFT pattern showing the [102] orientation. B) A higher magnification TEM image of the surface area showing a nanocrystal (NC) with corresponding FFT pattern consistent with Ta_3_N_5_.

**Figure 6:**
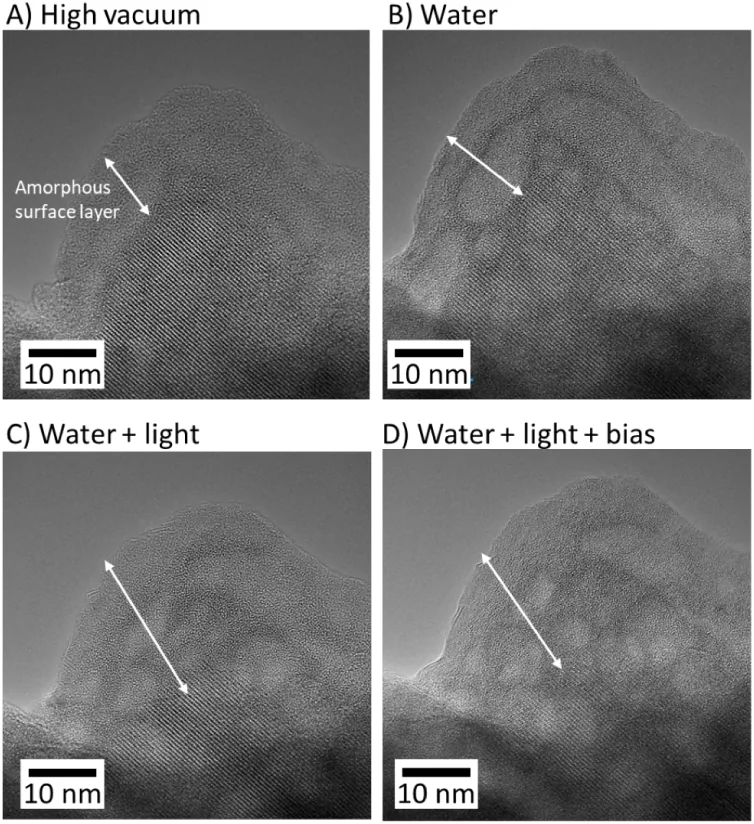
TEM images from the U-shaped FIB-prepared sample. The arrows indicate the amorphous surface layer. A) in high vacuum at the start of the experiment with additional carbon contamination layer on top, B) after exposure to 3 mbar of water vapor for 10 min, C) following exposure to both water vapor and light illumination for 10 min, and D) after exposure to water, light and 1.1 V bias for 10 min.

## Discussion

By combining ETEM with an *in situ* biasing holder, we enabled the simultaneous application of electrical bias, light illumination, and water vapor. The use of separate LED contact pads (Cu foil electrically isolated underneath) simplifies chip assembly, as only the sample requires FIB transfer to the E-chip. This configuration allows dynamic, real-time monitoring of Ta_3_N_5_ structural evolution under combined stimuli, confirming that photocorrosion is a synergistic process, with amorphization most pronounced when illumination, bias, and vapor are applied concurrently. The use of systematically varied single- and multistimulus conditions provides an internal set of control experiments, enabling isolation of the individual and synergistic contributions to degradation.

### Mechanistic Insights into Ta_3_N_5_ Degradation

Live recordings under combined stimuli with all three, reveal a three-step degradation pathway: (1) detachment of small Ta_3_N_5_ nanocrystals from the surface, followed by (2) their migration through the amorphous layer and (3) eventual amorphization and incorporation into the surrounding matrix (Figure 7). The apparent consumption of expelled nanocrystals by the amorphous phase indicates a structurally disordered and reactive interface that promotes atomic mobility and bond disruption. This results in an inward-propagating amorphization front, effectively “consuming” the crystalline core (schematic in Figure 7 and TEM images in Figure S7 in SI Section VI). Quantitative analysis (Figure 4) confirms that amorphous layer growth accelerates under combined stimuli, reinforcing the synergistic nature of degradation. Higher anodic driving forces, as encountered at potentials above ∼1.0 V vs RHE, are expected to enhance hole accumulation and interfacial oxidation, thereby promoting defect formation, nanocrystal detachment, and the inward propagation of amorphization observed here.

**Figure 7:**
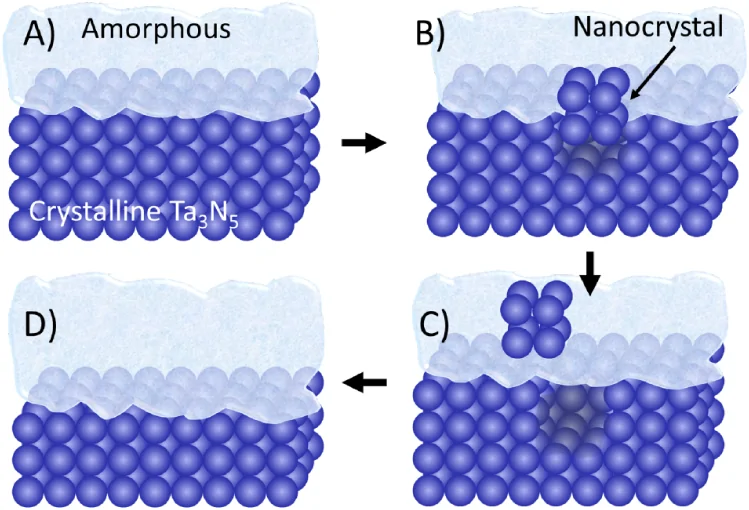
A) The crystalline Ta_3_N_5_ surface with a thin amorphous layer on top. B) Expulsion of a small crystalline nanoparticle from the surface. C) The nanoparticle becoming surrounded by the amorphous layer. D) Progressive amorphization of the nanoparticle as it is reabsorbed into the amorphous matrix.

This degradation pathway provides a structural basis for the formation of amorphous TaO_*x*_/Ta­(O,N)_*x*_ interphases, which are widely associated with reduced charge transfer and performance loss in Ta_3_N_5_ photoanodes. The observed mechanism therefore directly links nanoscale structural transformations to macroscopic device degradation.

This mechanism parallels degradation pathways observed in oxide-based oxygen evolution reaction (OER) catalysts, where electrochemically driven cation leaching and lattice oxygen evolution destabilize the lattice and promote amorphous layer formation.^25^ Although Ta_3_N_5_ is a nitride, the observed behavior aligns with this broader framework: defect accumulation and interfacial reactivity facilitate amorphization. Similarities also exist with solid-state corrosion and surface reconstructive dissolution, where amorphous phases act as sinks for mobile species and structural disordering.^26,27^ Beyond PEC materials, analogous amorphization phenomena occur in other systems, such as homogeneous amorphization of SiC nanoparticles under compressive strain.^28^ These parallels suggest that stress-assisted amorphization and surface reconstruction may be a general phenomenon across diverse material classes.

### Role of Geometry and Experimental Conditions

The more pronounced degradation observed in the U-shaped geometry (Figure 6) highlights the importance of lamella design during *in situ* experiments. Unlike the continuous Pt layer, the U-shaped configuration forces current to pass through the Ta_3_N_5_ material, creating localized potential gradients that strongly influence degradation behavior. If the lamella geometry does not mimic the current pathways present in an operating photoelectrode, the observed mechanisms may not reflect realistic PEC conditions. Careful control of sample geometry is therefore essential to ensure device-relevant insights. This simplified model system is intentionally designed to reveal intrinsic degradation mechanisms of Ta_3_N_5_, n practical photoelectrodes, protective coatings and cocatalysts can suppress or alter degradation processes, making it difficult to identify the underlying photocorrosion pathways. While such model systems may exhibit accelerated degradation compared to optimized devices, they provide direct access to the fundamental mechanisms governing photocorrosion.

### Design Implications for Stabilizing Ta_3_N_5_ Photoanodes

The degradation pathway identified here provides concrete guidance for the design of more stable Ta_3_N_5_-based photoelectrodes. First, the observed nanocrystal detachment and migration indicate that suppressing defect mobility at the crystalline–amorphous interface is critical, as mobile species facilitate structural breakdown and amorphous layer growth. Second, stabilizing the crystalline/amorphous interface itselfthrough reduced defect densities or interfacial passivation, may inhibit the inward propagation of amorphization. Third, the results demonstrate that degradation initiates locally at the crystalline–amorphous interface, suggesting that strategies aimed at reducing interfacial defect densities and suppressing localized structural instabilities may improve durability. Finally, strategies that prevent or hinder nanocrystal detachment, such as lattice stabilization, strain management, or defect engineering, may directly suppress the initiation of photocorrosion. Together, these design targets move beyond empirical stabilization approaches and establish a mechanistically grounded framework for improving the durability of Ta_3_N_5_ and related metastable PEC materials under operating conditions.

### Experimental Considerations and Beam Effects

Electron beam interactions in TEM can induce atomic displacement, sputtering, radiolysis, and electrostatic charging, potentially influencing electrochemical reactions.^29–32^ Beam-induced effects such as localized heating,^33^ defect formation,^34^ and electric field generation^35,36^ may amplify the applied stimuli, particularly in low-Z or insulating materials. Although amorphous layer growth was observed under both beam-on and beam-off conditions, beam contributions cannot be excluded. Future work should employ ultralow-dose or pulsed imaging, reduced acceleration voltages, or alternative modalities such as STEM with minimized dwell times to further isolate beam effects.

Overall, these findings highlight the importance of multistimulus *in situ* characterization for understanding PEC material degradation. By directly linking nanoscale structural transformations to macroscopic device failure, this approach enables rational, rather than purely empirical, development of stabilization strategies for next-generation photoanodes. While the absence of protective layers and cocatalysts may lead to accelerated degradation compared to optimized devices, this approach enables identification of the fundamental mechanisms governing photocorrosion.

## Conclusions

In this study, we employed ETEM combined with *in situ* biasing to investigate the degradation mechanisms of Ta_3_N_5_ photoanodes under simulated photoelectrochemical conditions. By integrating custom-fabricated chip-LEDs into an electrical biasing TEM holder, we enabled simultaneous exposure of the samples to water vapor, electrical bias, and light illumination. Our results demonstrate that Ta_3_N_5_ degradation is strongly governed by the combination of external stimuli, with concurrent application of all three leading to pronounced and accelerated surface amorphization.

*In situ* TEM imaging reveals a degradation pathway involving (1) expulsion of crystalline Ta_3_N_5_ nanocrystals from the amorphous–crystalline interface, followed by (2) their amorphization and incorporation into an expanding amorphous matrix. This process provides a structural origin for the formation of amorphous TaO_*x*_/Ta­(O,N)_*x*_ interphases, which are known to limit interfacial charge transfer and contribute to rapid photocurrent degradation in Ta_3_N_5_ photoanodes. The observed mechanism highlights a rate-dependent degradation pathway driven by the interplay of photoexcitation, electrochemical potential, and humidity. Targeting defect mobility and stabilizing the crystalline–amorphous interface therefore emerge as key strategies to inhibit nanocrystal expulsion and suppress photocorrosion.

This work underscores the importance of multistimulus *in situ* characterization techniques for directly probing dynamic transformations under operating conditions. The mechanistic insights obtained here not only advance the fundamental understanding of photocorrosion, but also establish concrete, mechanism-based design targets for improving the stability of Ta_3_N_5_ and related metastable photoelectrodes. More broadly, the approach presented here provides a framework for bridging nanoscale processes and macroscopic device degradation in complex energy materials.

## Materials and Methods

### Ta_3_N_5_ Films

Ta_3_N_5_ thin films were synthesized through a two-step process involving the deposition of metallic tantalum followed by thermal nitridation. Initially, Ta was deposited onto fused silica substrates using direct current (DC) magnetron sputtering in a nano-physical vapor deposition (PVD) system. The sputtering was conducted at room temperature under an argon flow of 10 sccm, with a DC power supply setting of 10% and a base pressure below 2 × 10^–6^ mbar. The deposition time was 60 min. Subsequently, the Ta-coated substrates underwent nitridation in an ammonia atmosphere at 950 °C for 8 h, employing a heating ramp rate of 10 °C/min to convert the metallic layer into Ta_3_N_5_.

### Characterization and FIB

The *ex situ* characterization of the sample was carried out using scanning electron microscopy (SEM) in a FEI Nova NanoSEM 650 instrument, and TEM imaging using a FEI Titan G2 TEM instrument equipped with DCOR Cs probe corrector, Super-X EDS detector and Gatan GIF Quantum 965 electron energy loss (EELS) Spectrometer at SINTEF in Oslo. The *in situ* TEM characterization was carried out using a Titan E-Cell 80-300ST ETEM at DTU in Denmark. The TEM samples for the *in situ* analysis were prepared using a Thermo Fisher plasma focused ion beam (PFIB)–SEM Helios Hydra instrument.

Figure S1 in SI Section I, shows some of the steps of the PFIB lift out process for Ta_3_N_5_ sample placed on E-chips. A 30 kV xenon ion beam was used for this purpose. The extracted slice was then lifted out and placed on the Protochips biasing E-chip and electrical connections were made between the E-chip’s electrodes and the sample by depositing *a carbon matrix with embedded Pt particles in situ*. The cutout slice was then thinned and polished to electron transparency. A 30 kV xenon ion beam with currents in the range 4 nA–30 pA was used during the thinning stage and the final polishing was done using an argon ion beam with energy 5 kV and current 60 pA. In case of the samples with U-shape geometry, a cut was made in the middle of the lamella, as shown in Figure S1F.

### LED Diode

To enable optical excitation of samples during TEM analysis, an InGaN-based white light-emitting diode (LED) was integrated directly onto the E-chip platform, see Figure S2 in SI Section II. The selected LED, the ASMT-CW00 (Broadcom/Avago Technologies), is a compact, side-emitting surface-mount device chosen for its small footprint (1.6 mm × 1.0 mm) and low profile (0.4 mm height), which allow close-proximity illumination within the spatial constraints of a TEM holder without interfering with the electron beam path or objective lens geometry. The ASMT-CW00 LED emits broad-spectrum white light with a typical luminous intensity of 35 mcd at a forward current of 5 mA and features a wide viewing angle of 170°, ensuring uniform illumination across the active sample area. It operates at a forward voltage between 2.55 and 3.15 V and supports continuous current up to 10 mA, with a maximum power dissipation of 30 mW.

For integration, the LED was soldered onto two strips of polyimide films with copper foil (35 μm, total thickness: 50 μm) (CIF Le Circuit Impremé Francais). The polyimide is insulating on one side, preventing electrical contact with the underlying chip. This assembly was then mounted onto an Electrical Fusion Select FIB-optimized E-chip (Protochips, Inc.), which features an open-slit design and recessed contact pads designed for precise lamella placement. In this configuration, the inner electrode channels were used to apply electrical bias to the lamella, while the outer electrode channels were used to power the LED. At an LED-sample distance of 3.5 mm, the maximum irradiance is therefore estimated to be 19.04 *W*/*m* ^2^. This estimate neglects losses from the holder geometry and assumes hemispherical emission; therefore, the true irradiance at the lamella may be lower.

### ETEM

The dynamic ETEM experiment was performed on a FIB-prepared sample with a carbon matrix with embedded Pt nanoparticles on Ta_3_N_5_. The sequence included: 1) imaging under vacuum to establish a baseline, 2) exposure to water vapor at 3 mbar for 10 min without electron beam illumination, 3) exposure to 3.10 mbar H_2_O­(g) while a 1.1 V bias was applied, 4) exposure to 2.9 mbar H_2_O­(g) combined with illumination from a light diode set to 3500 mV, and finally 5) a combined exposure at 3.6 mbar H_2_O­(g) with both a 1.1 V bias and 3500 mV light diode illumination for 10 min. The current and voltage profiles for the bias and diode are shown in Figure S3, corresponding to the experiment presented in Figure 5. The experimental sequence, involving systematic variation of water vapor, illumination, and electrical bias both independently and in combination, provides an internal set of control conditions that enables separation of the individual and synergistic contributions to degradation.

## Supplementary Material





## Notes

*In situ* TEM imaging under water, light, and bias conditions, showing nanocrystal detachment from the substrate and subsequent amorphization (MP4)
More details on the FIB preparation, ETEM setup, and analysis (PDF)
