# Supplementary material for: Direct Observation of Tantalum Nitride Photoelectrode Degradation by Environmental Transmission Electron Microscopy
Source: ACS Nanosci Au. 2026 Sep 26;6(5):1021–8. doi: 10.1021/acsnanoscienceau.6c00123 (PMC13692745; doi:10.1021/acsnanoscienceau.6c00123)
Supplement: Supplementary file 1 [file ng6c00123_si_002.pdf]

Supplementary Information for: Direct Observation of Tantalum Nitride Photoelectrode Degradation by Environmental Transmission Electron Microscopy.

Annett Thøgersen,[1] Shima Kadkhodazadeh,[2] Michael Stanislaus Seifner,[2] Monia Runge Nielsen,[2] Ingvild J. T. Jensen,[1] Martin Fleissner Sunding,[1] Ingeborg-Helene Svenum,[1] Mathieu Grandcolas,[1] Øystein Dahl,[1] Murat Nulati Yesibolati,[2] Thomas Willum Hansen,[2] and Athanasios Chatzitakis[3]

[1)] *SINTEF Industry, Materials Physics, Forskningsveien 1, 0373 Oslo, Norway*

[2)] *DTU Nanolab, Technical University of Denmark, Fysikvej, 2800 Kgs. Lyngby, Denmark*

[3)] *Centre for Materials Science and Nanotechnology, Department of Chemistry, University of Oslo, Gaustadalléen 21, NO-0349 Oslo, Norway*

(Dated: 25 September 2026)

I. FIB-PREPARATION

Fig. S1 shows the main steps in the focused ion beam (FIB) lift-out process. A thin slice was first cut and lifted out (Fig. S1A–B), then placed onto a Protochips biasing E-chip (Fig. S1C) at approximately 10° inclination angle. The slight inclination angle of the sample relative to the chip makes it possible to thin and polish both sides of the sample in the subsequent steps of the process. Electrical connections between the sample and the chip's electrodes were made by depositing platinum (Pt) *in situ* (Fig. S1D). The slice was then thinned and polished to electron transparency (Fig. S1E), using a 30 kV xenon ion beam with currents from 4 nA to 30 pA, followed by final polishing with a 5 kV argon ion beam at 60 pA. For samples with a U-shaped geometry, a cut was made in the middle of the lamella (Fig. S1F).

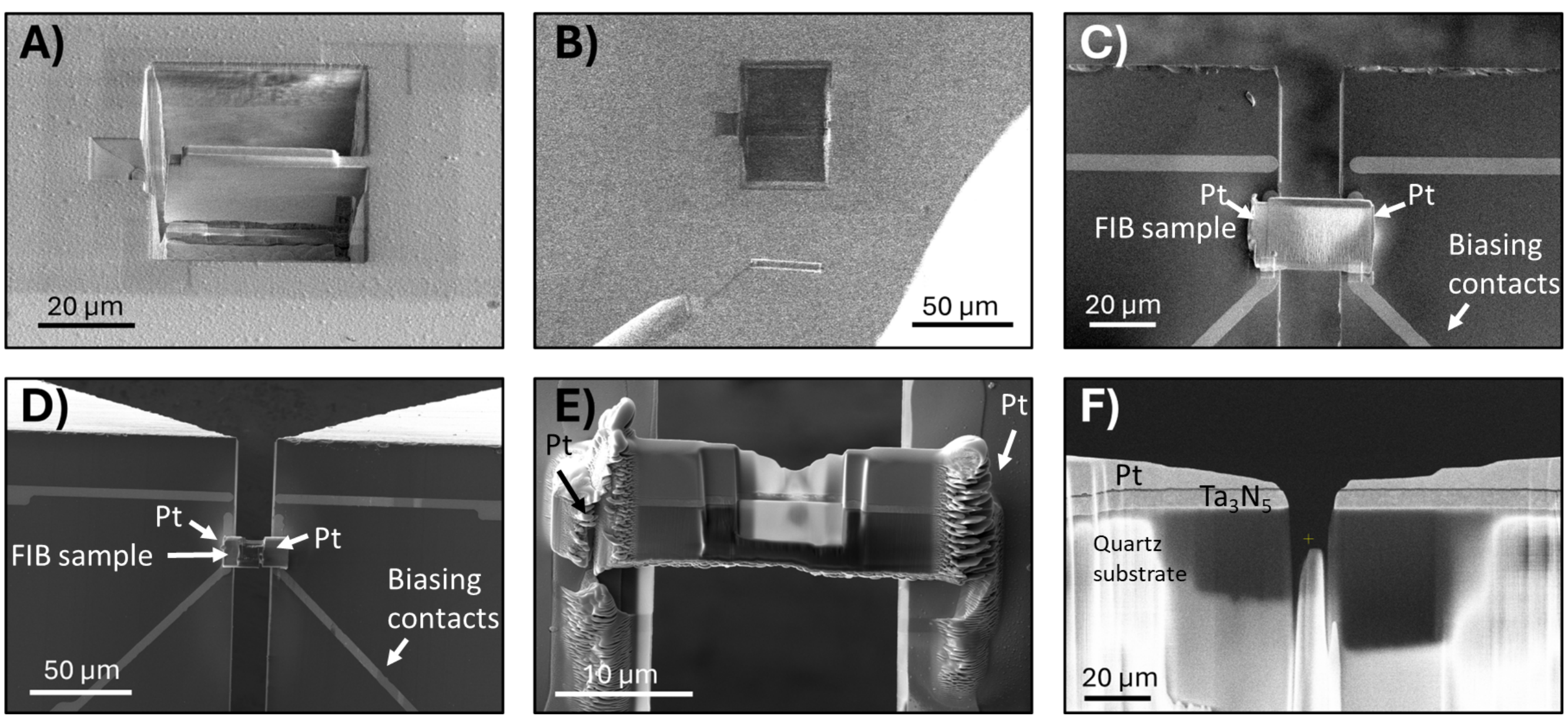

Fig. S 1: Sequential steps in FIB preparation of a U-shaped lamella: (A) initial milling of the Ta_3N_5 region, (B) lift-out of the lamella, (C) placement onto the Protochips biasing E-chip, (D) Pt deposition for electrical contacts and additional thinning and shaping on the chip, (E) fine milling and polishing of the sample, and (F) final lamella with U-shaped cut for controlled current pathways.

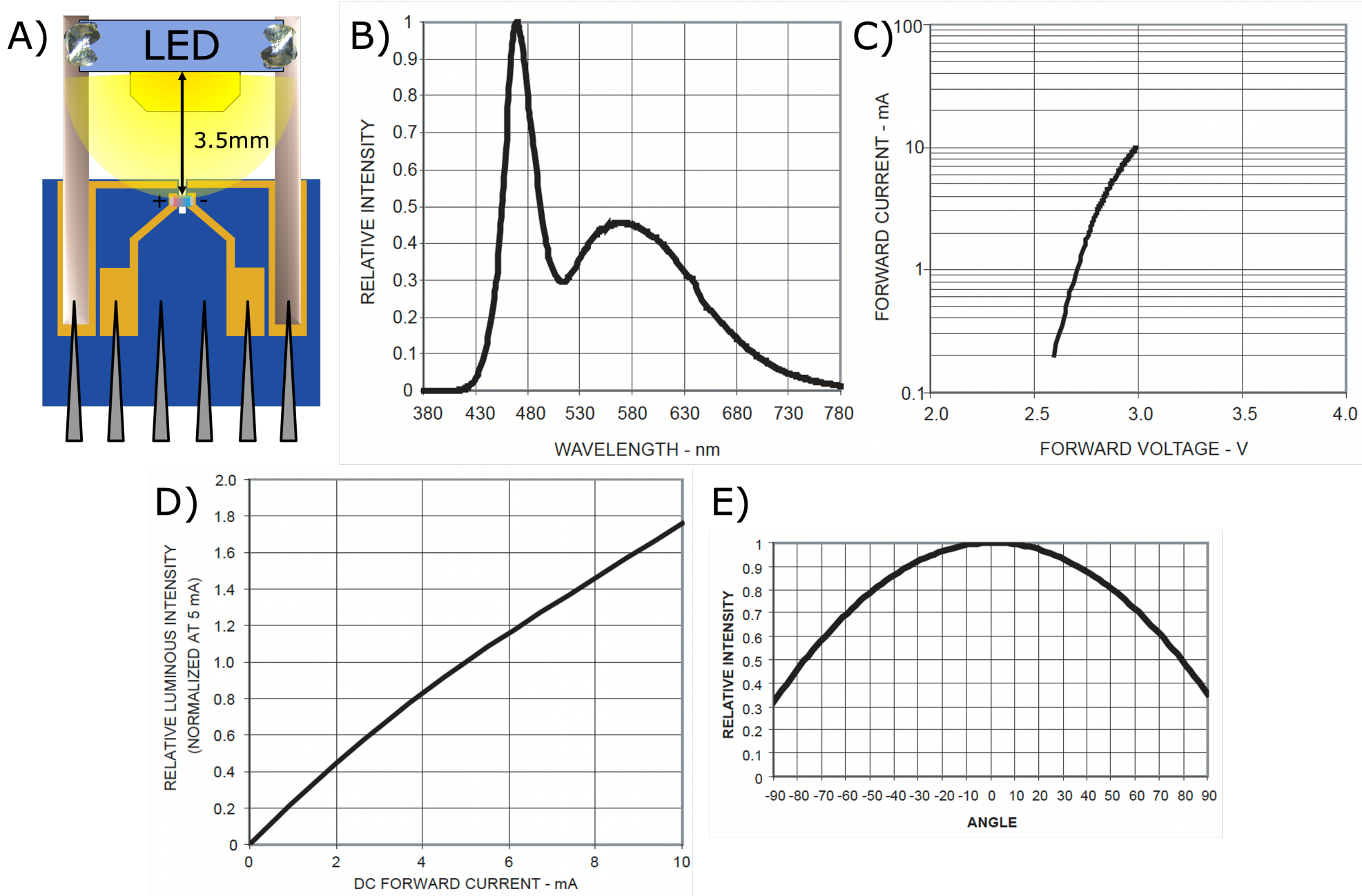

Fig. S 2: Characterization of the LED setup: (A) schematic of the diode integration on the TEM holder, (B) emission spectrum of the LED, (C) forward voltage as a function of current, (D) relative luminous intensity versus forward current, and (E) angular distribution of relative intensity. Data in B - C taken from[1]

II. ETEM-EXPERIMENT SETUP

Figure S2 shows the characterization of the LED setup, with the schematic of the diode integration on the TEM holder, (B) emission spectrum of the LED, (C) forward voltage as a function of current, (D) relative luminous intensity versus forward current, and (E) angular distribution of relative intensity.

A. Calculation of maximum light reaching the sample

The LED datasheet specifies a luminous intensity of 35 mcd at 5 mA. Figure S2D shows that the LED reaches 1.8×35 mcd $= 63$ mcd at 10 mA. However, the actual operating current during the experiment was 0.6 mA (Figure S3). Assuming a linear relationship between current and luminous intensity, the actual luminous intensity becomes:

$$I_v = 63 \text{ mcd} \cdot \frac{0.6 \text{ mA}}{10 \text{ mA}} = 3.78 \text{ mcd} \tag{1}$$

Convert luminous intensity to candela:

$$I_v = 3.78 \text{ mcd} = 3.78 \cdot 10^{-3} \text{ cd} \tag{2}$$

Assuming Lambertian emission, the total luminous flux is:

$$\Phi_v \approx 2\pi \cdot I_v = 2\pi \cdot 3.78 \cdot 10^{-3} \text{ cd} \approx 0.0237 \text{ lumens} \tag{3}$$

Convert luminous flux to optical power using a luminous efficacy of 300 lm/W (typical for InGaN white LEDs under optimal conditions):

$$P_{\text{opt}} = \frac{\Phi_v}{\text{efficacy}} = \frac{0.0237 \text{ lm}}{300 \text{ lm/W}} \approx 79\ \mu\text{W} \tag{4}$$

Assuming the LED radiates over a hemisphere and the sample is located $d = 3.5$ mm away, the surface area over which the light spreads is:

$$A = 2\pi d^2 = 2\pi \cdot (0.0035\ \text{m})^2 = 7.70 \cdot 10^{-5}\ \text{m}^2 \tag{5}$$

Note on luminous efficacy: The value of 300 lm/W refers to the *electrical* (wall-plug) luminous efficacy of high-performance InGaN white LEDs under optimal conditions, as reported in recent energy-efficiency benchmarks[2]. For simplicity, we assume near-unity conversion from electrical to optical power; deviations from this assumption, while possible, have a negligible effect on the estimated optical power due to the high internal quantum efficiency of modern LEDs.

The irradiance at the sample is then:

$$E = \frac{P_{\text{opt}}}{A} = \frac{79 \cdot 10^{-6}\ \text{W}}{7.70 \cdot 10^{-5}\ \text{m}^2} \approx 1.03\ \text{W/m}^2 \tag{6}$$

III. DYNAMIC ETEM EXPERIMENT

The bias and diode of the dynamic ETEM experiment of the FIB sample with a continuous Pt layer on Ta_3N_5 are shown in Figure S3, connected to the experiment in Figure 5 in the manuscript.

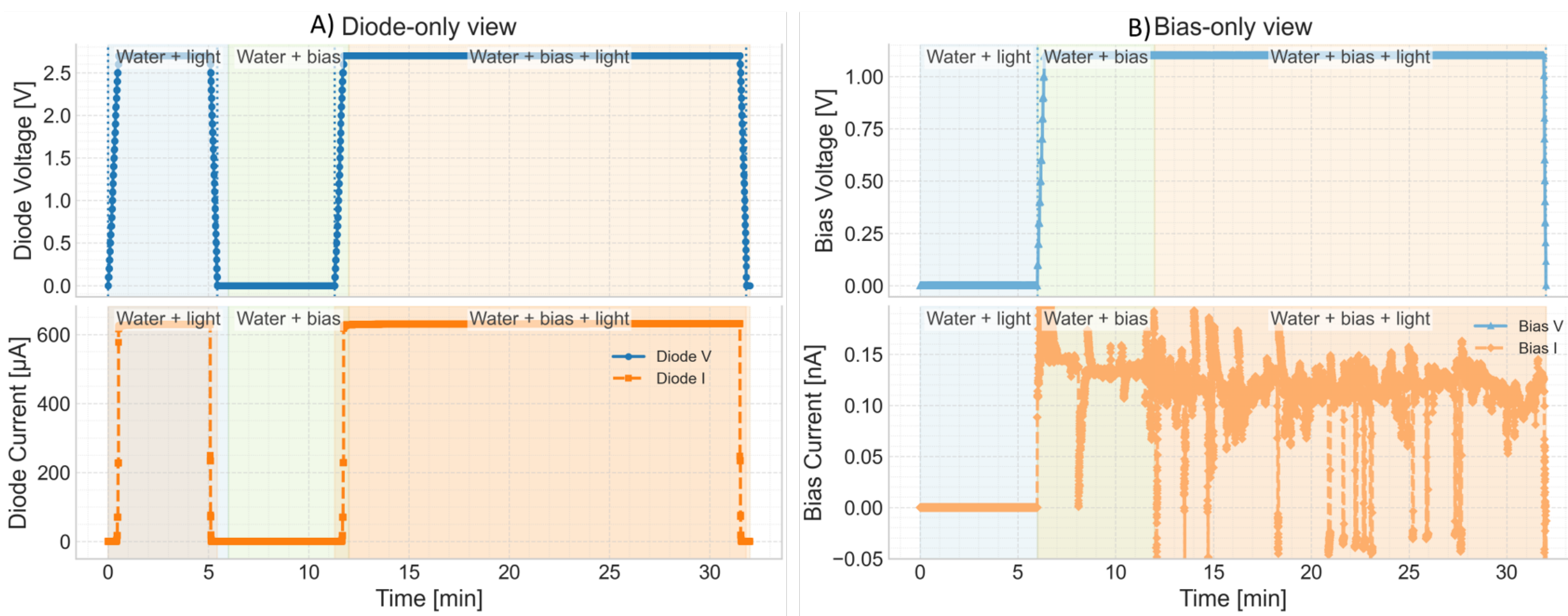

Fig. S 3: The voltage and current profiles of the dynamic experiment for A) The diode and B) Bias, during water + light, water + bias, and water + bias + light, where the blue curve corresponds to the voltage and the orange curves to the current.

IV. FFT ANALYSIS

FFTs of Figure 5 in the main manuscript show that there is no differences in the crystallographic orientations in this area of the sample after exposing the sample to water vapor, bias and light, see Figure S4.

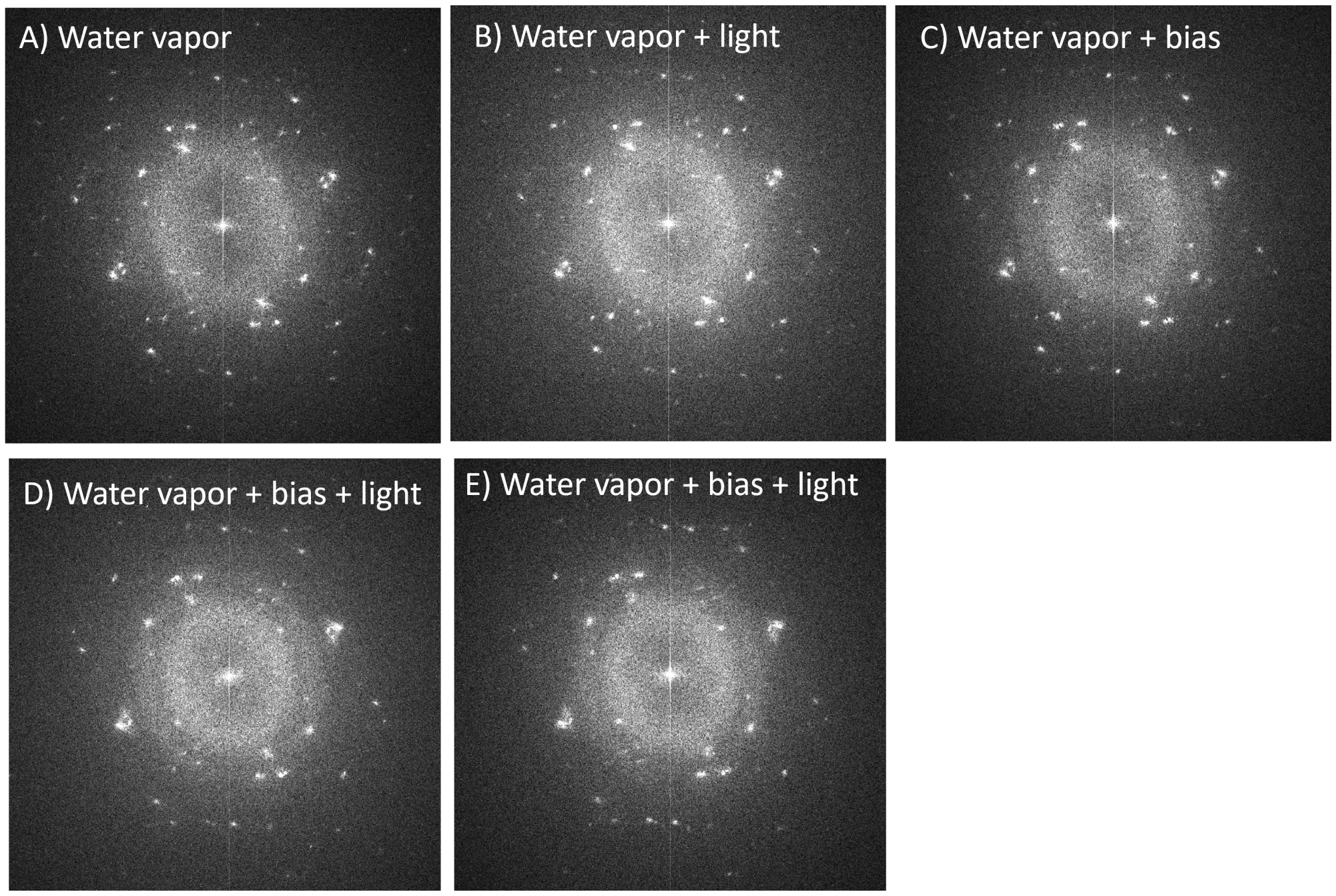

Fig. S 4: FFTs of images of the Ta_3N_5 surface in Figure 7 after being exposed to: A) water vapor, B) water vapor and light, C) water vapor and bias, D) water vapor, bias, and light, and E) water vapor, bias after 1 minute, and light in the end of the experiment.

V. SURFACE AREA CALCULATIONS

In order to estimate the growth of the amorphous layer during the experiment, the images recorded at different stages were segmented and the surface area of the amorphous layer in each case was calculated. The segmentation was carried out manually using the 'Image segmenter' app in MATLAB. The segmented binary images are shown in Figure S5 and Figure S6.

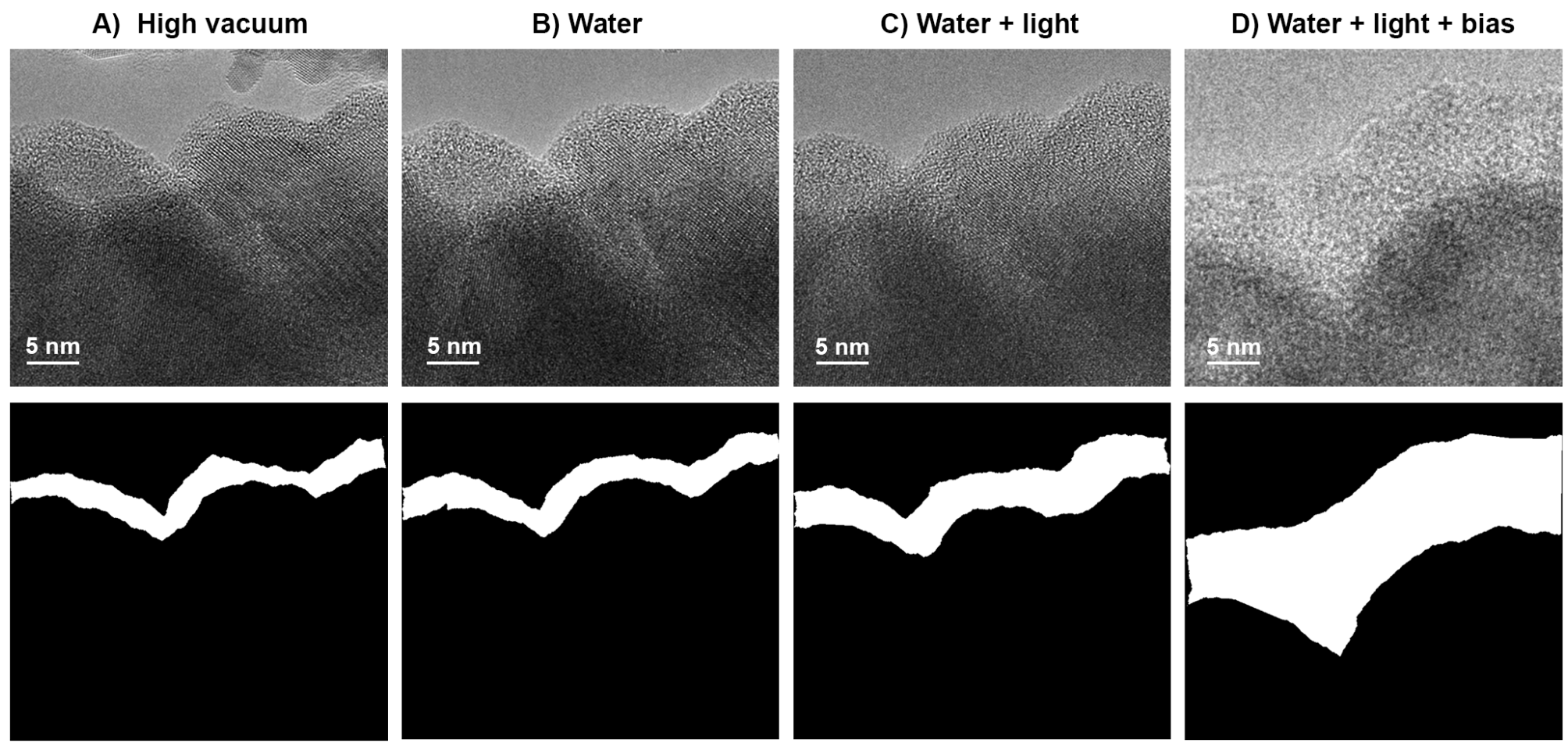

Fig. S 5: Original (first row) and segmented (second row) TEM images of the same region of the sample A) in high vacuum, and after exposure to B) water, C) water and light illumination, and D) water, light and electrical bias. The sample was not imaged in TEM while these stimuli were applied (beam off). See Figure 3 in the main manuscript for further details related to the experiment.

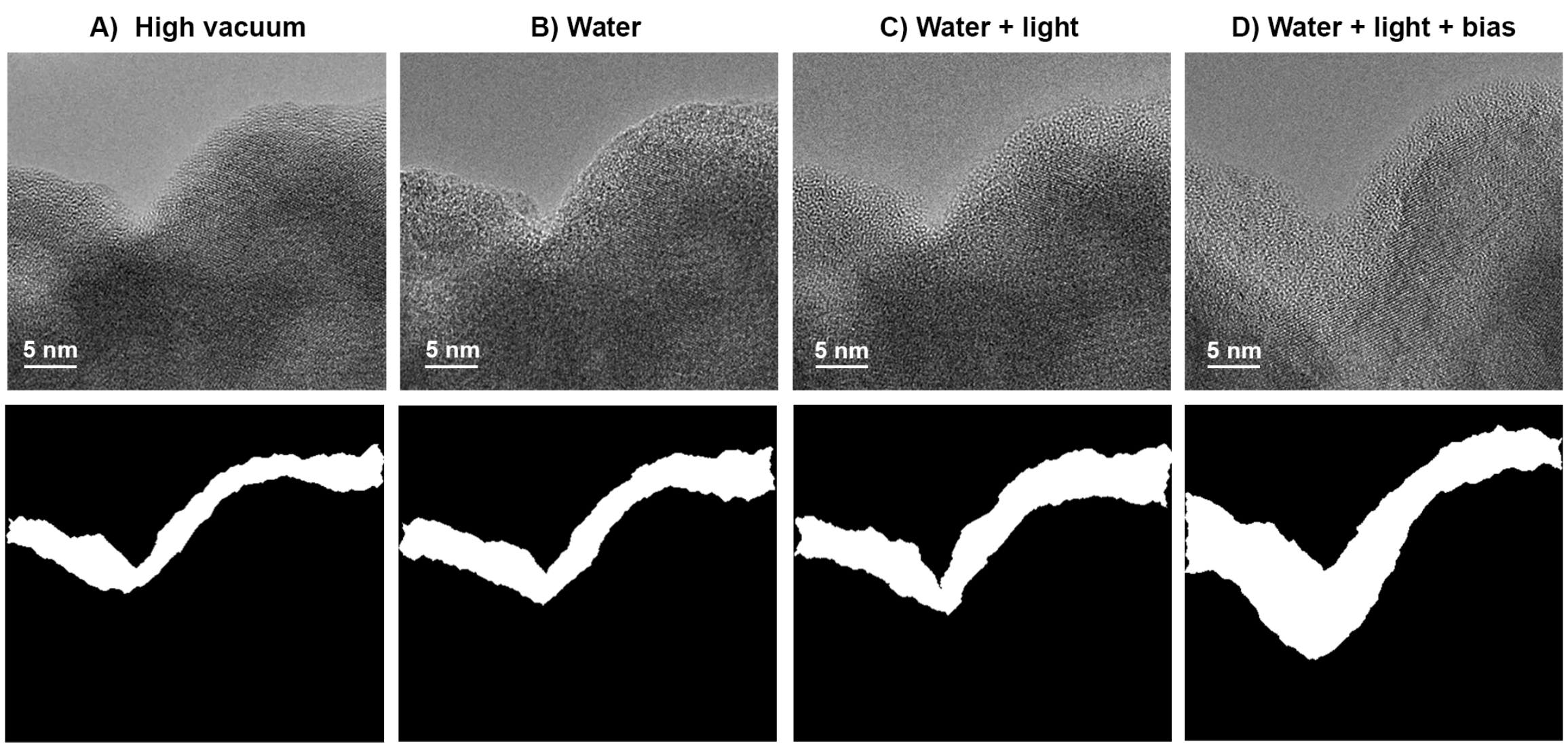

Fig. S 6: Original (first row) and segmented (second row) TEM images of the same region of the sample A) in high vacuum, and after exposure to B) water, C) water and light illumination, and D) water, light and electrical bias. The sample was imaged continuously in TEM while these stimuli were applied (beam on). See Figure 3 in the main manuscript for further details related to the experiment.

VI. NANOCRYSTAL EXPULSION AND AMORPHIZATION

Figure S 7 shows nanocrystals move from the Ta_3N_5 sample surface and into the amorphous surface layer, for then to become amorphous after reaching the surface.

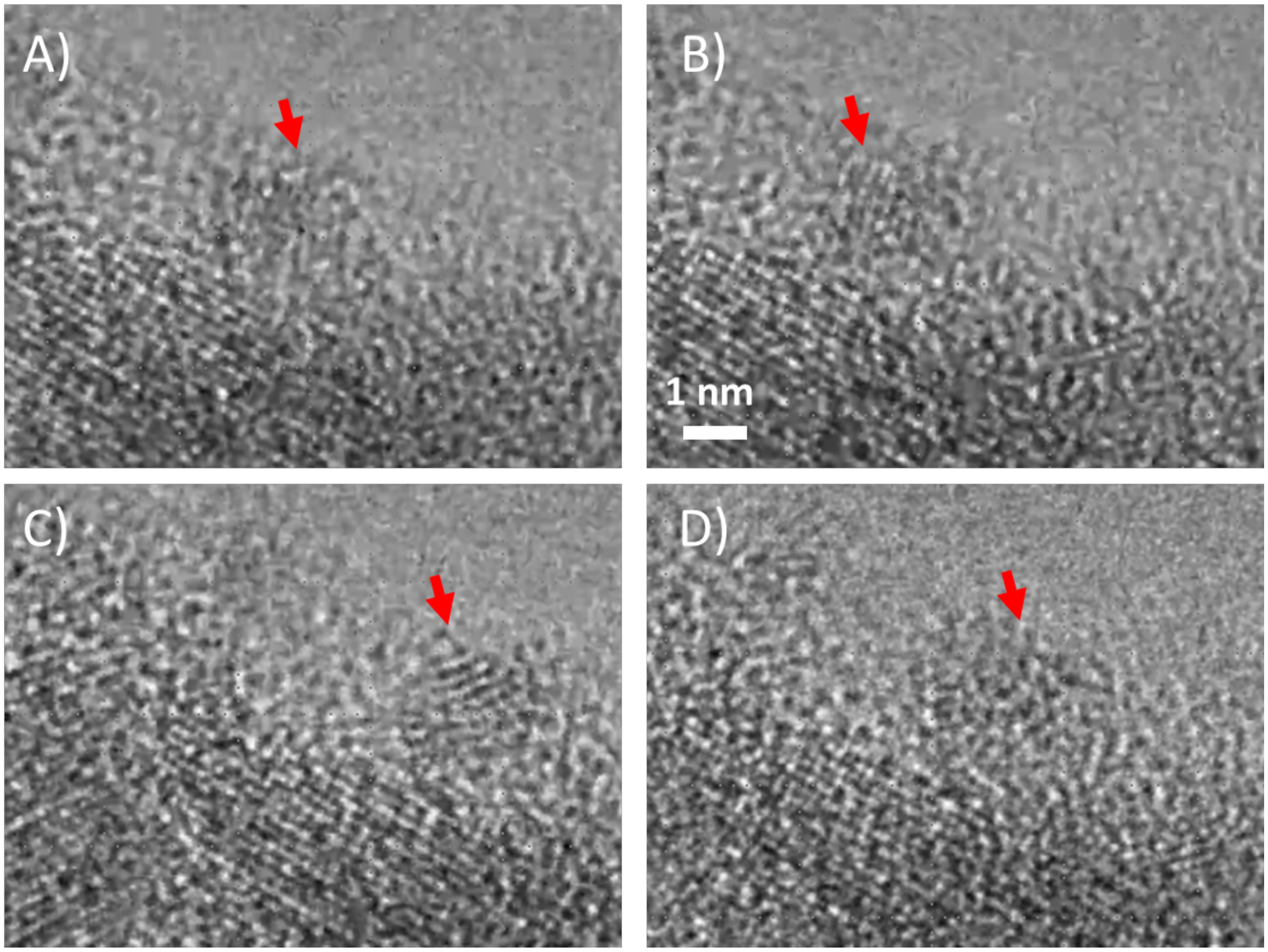

Fig. S 7: TEM images showing the sequential degradation of the Ta_3N_5 surface: A) initial crystalline state, B) onset of disruption, C) nanocrystal expulsion, and D) amorphization and reabsorption into the surface layer.

REFERENCES

[1] "Broadcom asmt-cw00 right angle chip led (ingan based white led)," (2019), broadcom ASMT-CW00 Right angle Chip LED (InGaN based white LED), https://www.broadcom.com/products/leds-and-displays/chip-leds/right-angle-surface-mount/asmt-cw00.

[2] U.S. Department of Energy, "Energy efficiency of leds," https://www1.eere.energy.gov/buildings/publications/pdfs/ssl/led_energy_efficiency.pdf (2012).
